# Misdiagnosis of inclusion body myositis: two case reports and a retrospective chart review

**DOI:** 10.1186/s13256-015-0647-z

**Published:** 2015-08-13

**Authors:** Amaiak Chilingaryan, Richard A. Rison, Said R. Beydoun

**Affiliations:** 1 Neuromuscular Division, Keck School of Medicine, University of Southern California, 1520 San Pablo Street, Suite 3000, Los Angeles, CA 90033 USA; 2 Keck School of Medicine, University of Southern California, Los Angeles County Medical Center, PIH Health Hospital-Whittier Stroke Program, 12401 Washington Boulevard, Whittier, CA 90602 USA

**Keywords:** Amyotrophic lateral sclerosis, Misdiagnosis, Sporadic inclusion body myositis

## Abstract

**Introduction:**

Sporadic inclusion body myositis is the most common adult myopathy in persons aged 50 years and older. The clinical presentation includes a chronic, slowly progressive course with a predilection for weakness of the forearm flexors and quadriceps muscles. Its indolent course makes it a disease frequently missed or misdiagnosed as other neuromuscular conditions by health care professionals. The degenerative processes with amyloid accumulation distinguish sporadic inclusion body myositis from other inflammatory myopathies. Currently, no effective therapy exists. This clinical report highlights the difficulties in diagnosing the disease, examples of misdiagnosis, and inappropriate therapies that can result from misdiagnosis.

**Case presentation:**

We present our clinical experience with 20 patients over a 10-year period and describe in depth two cases, both men, one of Indian ethnicity and the other of Hispanic ethnicity, who were referred to our neuromuscular division for second opinions and diagnosed with sporadic inclusion body myositis years after symptom onset.

**Conclusions:**

Although sporadic inclusion body myositis is rare and without effective therapy, accurate diagnosis is crucial to providing adequate counseling and information about the prognosis and disease course, and to avoiding inappropriate therapy.

## Introduction

Sporadic inclusion body myositis (s-IBM) is one of several chronic adult inflammatory myopathies. Its prevalence varies, but it may be as high as 35 per 1 million adults over age 50 years, with a slight male predominance [1]. The clinical presentation involves chronic, slowly progressive, distal asymmetric weakness affecting the finger flexors and proximal lower extremity weakness affecting the quadriceps, which later progresses to other proximal and distal muscles. The indolent disease course sometimes lasts many years until patients notice significant deterioration leading to medical care [2–4]. Additional complaints include dysphagia caused by cricothyroid muscle weakness and decreased pharyngeal propulsion [5]. The pathogenesis of s-IBM is not fully understood, and currently there is no known treatment [3, 6]. We present two exemplary cases followed by a summary table that highlights the presentation of 20 patients seen in our clinic over a 10-year period.

## Case presentations

The local institutional review board approved this study. The case presentation portions of this report were prepared according to recent standardized guidelines [7–9].

### Patient 1

A 58-year-old man of Indian ethnicity with type 2 diabetes mellitus was referred to our hospital for a second-opinion neuromuscular evaluation. Four years prior, he had noted leg weakness, particularly in his thighs. After initial evaluation and diagnostic studies, his neurologist had diagnosed him with deconditioning and prescribed vitamin B_12_ injections. A subsequent electrodiagnostic study revealed denervation changes in his upper extremity muscles and thoracic paraspinal muscles, leading to a diagnosis of amyotrophic lateral sclerosis (ALS), which the patient carried for nearly 1 year. He was prescribed riluzole and advised to plan for end of life.

When referred to our institution, the patient described weakness while climbing and descending stairs without upper extremity complaints. During his physical examination, he was noted to have atrophy in the quadriceps muscles with moderate weakness. Electromyography (EMG) showed increased membrane instability and early recruitment with fractionation of the motor unit potentials, showing a brief, small, abundant, polyphasic motor unit potential pattern. His creatine kinase (CK) level was elevated at 647IU/L. A muscle biopsy showed myopathic features with rimmed vacuoles characteristic of s-IBM. The patient was informed of his correct diagnosis and counseled on his prognosis. Patient 1 is represented as patient 8 in Table 1.

**Table 1:** Retrospective chart review

Patient	Age (yr)	Time to diagnosis (mo)	Sex	Symptoms	Examination at initial presentation^a^	Electrodiagnostic studies	Muscle biopsy	Diagnosis before referral
1	56	24	M	Gait difficulty, then grip weakness	WE 4− (L), FF 2, HF 5− (L), KE 3, DF 2	Myopathic	End-stage muscle wasting	Possible myopathy
2	70	60	M	Asymmetric leg weakness, recurrent falls	HF 4− (L), HE 4−; HF 3 (R), KE 4−, KF 5−, DF 4,	Myopathic	s-IBM strongly confirmed by p62-positive fibers	Unknown
3	76	36	M	Difficulty getting up from chair then difficulty opening jars; mild dysphagia	SA 5−, EF 4−, EE 4−, FE 4; digits 4 and 5 FF 4 (L), HF 4−, KE 4	Mixed proximal myopathy	End-stage muscle wasting, no inclusion bodies or lymphocytic infiltrates	Probable myopathy
4	81	48	M	Leg weakness, falls, difficulty getting up from chair	Mild digit 5 flexion weakness, HF 4	Myopathic	Fibers with adjacent lymphocytes, inclusions in fibers, interstitial fibrosis, COX-negative fibers	Comorbid RA
5	85	72	M	Difficulty climbing stairs, then asymmetric grip weakness	Distal FF 2, KE 3, PF 4+ (L)	Length dependent axonopathy, myopathy	Atrophy with scattered morula, with mild chronic inflammation	Peripheral neuropathy
6	67	60	M	Grip weakness, then difficulty getting up from chair	FF 3, HF 3, KE 2, PF 4	NA	Intramyofiber inclusions	Unknown
7	77	48	F	Getting up from chair and gait difficulty, then difficulty opening jars	SA 2, EF 2, EE 4, FF 2, FE 4+, HE 4, HA 4, KE 2, KF 4−, DF 4−, PF 4−	Myopathic	NA	Unknown
8	55	48	M	Proximal leg weakness, difficulty getting up from chair, climbing stairs	FF 4, HF 5−, HE 5−, KF 4−, KE 4	Myopathic	Chronic and active vacuolar myopathy, severe IBM	ALS
9	54	145	M	Grip weakness, then leg weakness, getting up from chair and ambulation difficulty	FF 3 (R); 4 (L), KE 3 (L), KE 4− (R)	Myopathic	2003: polymyositis	Polymyositis
							2013: chronic and active vacuolar myopathy	
10	68	48	M	Weakness with grip, opening jars, handling coins, then proximal leg weakness	EF 5−, EE 4+, FF 3 (R) 2 (L), KE 4− (R); 2 (L), HF 4+	Myopathic	Intramyofiber inclusions with fibrosis, absent oxidative positivity	Neuropathy vs. myopathy
11	63	36	F	Proximal leg weakness, then finger flexor weakness, some difficulty swallowing solids.	Digits 4 and 5 FF 2 (R); digits 2–5 FF 2 (L), KE 4−, DF 4+ (R); 4− (L), PF 4+	Myopathic	Prominent lymphocytic infiltration	Unknown
12	73	120	M	Proximal leg weakness	HE 4, KE 3	Chronic denervation in the L4 muscles	Biceps with minimal denervation changes, quadriceps too atrophic to biopsy	Possible myopathy
13	43	60	M	Finger flexor and proximal leg weakness	HE 4−, KE 3+ (R); 4− (L)	NA	Lymphocytes, myopathy	Inflammatory myopathy vs. IBM
14	67	24	M	Proximal leg weakness, then finger flexors	HF R 4, KE R 4 (R); 4+ (L), DF R 2 (R); 4+ (L)	Myopathic	Multifocal endomysial inflammation and fibrosis, intramyofiber inclusions	Extrapyramidal disease vs. lumbosacral radiculopathy
15	75	6	M	L leg weakness, L hand weakness, b/l arm weakness	SA 4, FF 4−, HF 3, HA 4, KF 4+, DF 4+, KE 4−	Myopathic	Atrophic myofibers, minimal endomysial inflammation, rimmed vacuoles	Unknown
16	71	120	F	Leg weakness, then hand weakness, then difficulty swallowing	B 4+ (R); 4 (L); WE 4 (R), FE 3, FF 3, KE 4−	Myopathic	Intramyofiber inclusions	IBM
17	81	240	F	Proximal leg weakness, then finger flexor weakness; some difficulty swallowing	SA 3 (R); 4− (L), FE 4−, WE 4−, WF 4−, FF 4−, HF 2, HE 3, KE 3, DF3, PF 3	NA	Severe inflammatory myopathy	Polymyositis
18	82	24	M	R hand weakness, then L hand weakness	FF 3R (R); 2 (L)	Myopathic	Ragged red fibers, COX-negative fibers	ALS
19	56	60	M	Progressive weakness in hand grip	WF 5−, FF 4	Myopathic	NA	Possible ALS
20	55	120	M	Difficulty holding heavy objects, then difficulty getting up from chair	EE 4, FF 4, HF 4−, KE 5−	NA	Endomysial mononuclear infiltration, internal nuclei in many fibers, ragged red fibers with rimmed vacuoles, COX-negative fibers	IBM

*Abbreviations*: *ALS* amyotrophic lateral sclerosis, *B* biceps, *b/l* bilateral, *COX* cytochrome oxidase, *DF* dorsiflexors, *EE* elbow extension, *EF* elbow flexion, *FE* finger extension, *FF* finger flexion, *HA* hip abduction, *HE* hip extension, *HF* hip flexion, *IBM* inclusion body myositis, *KE* knee extension, *KF* knee flexion, *L* left, *LE* lower extremity, *NA* not available, *PF* plantarflexors, *pt* patient, *PT* physical therapy, *R* right, *RA* rheumatoid arthritis, *SA* shoulder abduction, *s*-*IBM* sporadic inclusion body myositis, *UE* upper extremity, *WE* wrist extension, *WF* wrist flexion

^a^Strength is given according to the Medical Research Council grading system. Muscle groups not listed are otherwise normal strength

### Patient 2

A 54-year-old Hispanic man developed weakness in his grip, followed 1 year later by lower extremity weakness. His CK level was 2400IU/L, and a muscle biopsy indicated polymyositis (PM). His initial therapy included prednisone followed by other immunosuppressive medications, including azathioprine, methotrexate, and mycophenolate mofetil. Although the patient’s CK level decreased to 450IU/L, his condition continued to progress. Nine years later, he was evaluated at our neuromuscular clinic. At that time, he was unable to get up from a seated position, locked his knees while ambulating to avoid falls, and had severe weakness in his left hand and moderate weakness in his right hand. During his physical examination, he was noted to have asymmetric atrophy in the forearm flexor and the quadriceps muscles, with severe asymmetric weakness of knee extension and slight weakness in ankle dorsiflexion and plantarflexion. EMG showed active denervation with significant brief, small, abundant, polyphasic motor unit potentials. A repeat muscle biopsy showed variation in muscle fiber size, endomysial fibrosis, chronic inflammatory cells with macrophages, and rimmed vacuoles with basophilic stippling, all characteristic of s-IBM. The patient was counseled on his correct s-IBM diagnosis, and immunosuppressive therapy was discontinued. Patient 2 is represented as patient 9 in Table 1.

## Retrospective chart review

Table 1 shows 20 patients (16 men, 4 women) seen at our neuromuscular division between 2004 and 2014 (including patients 1 and 2 in the present report listed as patients 8 and 9, respectively). The patients’ average age at initial evaluation at our clinic was 67.8 years (standard deviation, 11.5; range, 43–85). The average number of months from symptom onset to diagnosis was 70.0 (standard deviation, 54.8; range, 6–240). One patient did not follow up to confirm the diagnosis but was clinically diagnosed with s-IBM. Our clinic population had characteristics of s-IBM similar to those described in previous reports. The topography was proximal leg weakness presenting as difficulty with ambulation and rising from chairs, in addition to finger flexor weakness presenting as difficulty handling objects and opening jars and bottles. Four patients had dysphagia.

Several patients were misdiagnosed before evaluation at our institute: two patients (patients 9 and 17) diagnosed with PM who were given immunosuppressive medications; three (patients 8, 18, and 19) who were diagnosed with ALS; one (patient 5) diagnosed with entrapment neuropathy who underwent cubital tunnel release with no improvement; one (patient 14) who was diagnosed with extrapyramidal symptoms of Parkinson’s disease versus radiculopathy; and one (patient 16) who was diagnosed with s-IBM and given etanercept. The remaining 12 patients were referred without diagnosis or for second opinions for myopathy.

(Please note the standard deviations given above for time until the final correct diagnosis, and see below for discussion of trends and how our findings coincide with the existing published literature.)

## Discussion

s-IBM is both a myodegenerative and neurodegenerative disease with β-amyloid-related deposits and an inflammatory disease with endomysial lymphocytic infiltration. Accumulation of misfolded proteins and inadequate intracellular repair mechanisms characterize the disease [10]. There is also expression of major histocompatibility complex (MHC) class I molecules [3]. A 43 kDa muscle protein, recently identified as cytosolic 5′-nucleotidase 1A, was discovered in 2013 as an autoantigen for s-IBM autoantibodies [11–13]. This finding indicates the potential role of humoral immunity in the pathogenesis of the disease in addition to a cytotoxic T-cell-mediated component. Furthermore, identification of this target of s-IBM autoantibodies could lead to the development of a diagnostic blood test, potentially eliminating the need for invasive testing such as muscle biopsy.

Although muscle biopsy can confirm the diagnosis of s-IBM, the results may be negative owing to sampling error or to end-stage muscle wasting. Endomysial inflammation and degeneration with misfolded protein aggregates and/or inclusions [10] and atrophic fibers [14] are often present. Congo red staining or immunofluorescence shows amyloid β-pleated sheets in and around vacuoles [3]. There are rimmed vacuoles within the myofibril. In cases where the inclusion bodies are not found, the presence of inflammation alone can lead to an erroneous diagnosis of PM [4, 15]. If there is no definite diagnosis, then biopsy should be repeated.

Currently, there is no cure for s-IBM. Some patients may initially show a limited response to corticosteroids. Other treatments available for inflammatory myopathies have efficacy in s-IBM in specific cases [3, 16]. Patients with dysphagia may benefit from intravenous immunoglobulin (IVIG) therapy, along with balloon dilation or botulinum toxin injection [16–20]. Although this disease is rare and incurable, making the correct diagnosis is crucial to directing the patient to physical therapy for weakness, gait training, and education to prevent falls. Occupational therapy may improve a patient’s ability to engage in activities of daily living. Appropriate patients with swallowing complaints should be referred to a speech therapist for proper education regarding diet consistency and aspiration precautions. Patients may be assured that s-IBM is not a motor neuron disease or a rapidly deteriorating myopathic condition that is life-threatening [6].

Our university neuromuscular clinic sees approximately six new patients with s-IBM each year. The median age of the predominantly male population in our present report was 69 years, and patients’ ages ranged from 43 to 85 years. The median time from symptom onset to final diagnosis was 54 months and ranged from 6 months to 20 years, reflective of the slow disease course that helps to differentiate s-IBM from other disease mimickers. Experienced neurologists and neuromuscular specialists can diagnose s-IBM based on its topography of weakness of quadriceps and forearm flexors, but the diagnosis is often missed, delayed, or incorrect. Common misdiagnoses are PM, immune-mediated neuromuscular disease, entrapment neuropathies, and motor neuron disease. Erroneous diagnosis can lead to inappropriate therapy, as illustrated by some of the cases described here and in a previous report of patients who received years of corticosteroids and immunosuppressive medication and experienced serious side effects [6]. IVIG was previously used for treatment of s-IBM until a controlled clinical trial showed lack of efficacy [2]. Needless surgical treatments for radiculopathy or entrapment neuropathy have occurred, as illustrated by patient 5 in our series.

Although s-IBM is a well-recognized neuromuscular diagnosis that is seen in neurology and neuromuscular specialty clinics, it is well known that it can masquerade as other disorders [6]. The cases described here confirm and highlight the fact that s-IBM is still difficult to diagnose and remains frequently misdiagnosed. Our data show that the time to diagnosis averaged 5.83 years, a delay similar to that described by Lotz and coworkers in 1989 [21]. The fact that this has remained unchanged for 25 years is disappointing but not entirely surprising, given the symptoms and neurological topography, including asymmetric weakness, finger flexor weakness, loss of grip strength, gait difficulty, and dysphagia, that overlap with those of non-s-IBM conditions. The electrodiagnostic findings in our clinical neurophysiology laboratory in this case series also coincide with previous reports of mixed patterns, which may confuse non-neuromuscular clinicians.

We find several pitfalls in the diagnosis of s-IBM. First is an overreliance on electrophysiology. Second, muscle biopsies sometimes do not have all the cardinal histological features, including endomysial inflammation, newer findings of MHC class II upregulation and invasion of non-necrotic muscle fibers by lymphocytes, and mitochondrial changes [22–24]. Also, clinicians may be misled by an incomplete biopsy appearance, with patchy inflammatory changes being more florid early and patchy degenerative changes more florid later in the disease course. A third pitfall arises when patients present early with either atypical symptoms, such as camptocormia or foot drop, or an incomplete clinical picture. Clues to look for in a clinical examination include long finger flexor and quadriceps weakness, as confirmed in our present study. It has been suggested that s-IBM can be made by clinical diagnosis alone [25]. To the general practice clinician, however, we recommend that all cases of suspected s-IBM be referred to a center specializing in neuromuscular disease so that an appropriate diagnosis can be made and inappropriate treatments are avoided.

Limitations of our study include the relatively small number of patients. Our present study of 20 patients is only half the number of the series previously reported by Lotz and colleagues (40 patients) [21]. Also, our study may have university and/or tertiary geographic referral bias, along with limitations of our single-center experience. We do hope that our aforementioned clinical points are clear, however, regarding potential misdiagnosis and the importance of correct diagnosis.

## Conclusions

s-IBM is still difficult to diagnose and unfortunately remains frequently misdiagnosed, with a delay in accurate diagnosis of just over 5½ years, which has not changed over the last 25 years. Although s-IBM is rare and without effective therapy, accurate diagnosis is of crucial importance to providing adequate patient counseling and information about the prognosis and course of the disease. Cases of suspected s-IBM should be referred to a center specializing in neuromuscular disease so that an appropriate diagnosis can be made and inappropriate treatments are avoided.

## Consent

Written informed consent was obtained from both of the patients for publication of this case report and accompanying images. A copy of the written consent is available for review by the Editor-in-Chief of this journal.

## Abbreviations

ALS: Amyotrophic lateral sclerosis
b: Biceps
b/l: Bilateral
COX: Cytochrome oxidase
CK: Creatine kinase
DF: Dorsiflexors
EE: Elbow extension
EF: Elbow flexion
EMG: Electromyography
FE: Finger extension
FF: Finger flexion
HA: Hip abduction
HE: Hip extension
HF: Hip flexion
IBM: Inclusion body myositis
IVIG: Intravenous immunoglobulin
KE: Knee extension
KF: Knee flexion
LE: Lower extremity
MHC: Major histocompatibility complex
PF: Plantarflexors
PM: Polymyositis
pt: Patient
PT: Physical therapy
R: Right
RA: Rheumatoid arthritis
SA: Shoulder abduction
s-IBM: Sporadic inclusion body myositis
UE: Upper extremity
WE: Wrist extension
WF: Wrist flexion
